# Supporting diversity in clinical trials: the equitable breakthroughs in medicine site maturity model

**DOI:** 10.1186/s13063-024-08594-9

**Published:** 2024-11-14

**Authors:** Tesheia Harris, Marcella Nunez-Smith, Sakinah C. Suttiratana, Samantha L. Fretz, Savannah Leonard, Erika Linnander, Leslie A. Curry, and the EQBMED Network Partners

**Affiliations:** 1 Department of Internal Medicine, Yale Center for Clinical Investigation, Yale School of Medicine, New Haven, CT USA; 2 Department of Internal Medicine, Equity Research and Innovation Center, Yale School of Medicine, New Haven, CT USA; 3 Global Health Leadership Initiative, Yale School of Public Health, 60 College Street, New Haven, CT 06510 USA

**Keywords:** Clinical trial diversity, Community engagement, Maturity assessment model

## Abstract

**Background:**

Among the most powerful barriers to broader inclusion of diverse participants in clinical trials are social determinants of health, trustworthiness of health care providers and research institutions, and competing pressures on potential participants. Nevertheless, current tools to assess organizational capabilities for clinical trial diversity focus primarily on trial infrastructure, rely solely on quantitative self-reported data, and lack meaningful assessment of capabilities related to community engagement.

**Methods:**

The Equitable Breakthroughs in Medicine (EQBMED) initiative developed a holistic, collaborative, site-driven formative model and accompanying assessment to catalog sites’ current capabilities and identify opportunities for growth in both conducting industry-sponsored clinical trials and enriching diversity of those trials. The model builds upon prior work and reflects unification of two historically distinct components—research operations and community engagement—since sustainable clinical trial diversity efforts must overcome these silos. Here we present the methodology we used to develop the model and accompanying assessment, describe how findings can support clinical trial diversity efforts, and report findings from early field testing at three U.S. sites.

**Results:**

The first three sites were diverse in size (e.g., < 250–1 K beds), with varying levels of clinical trial capabilities and community engagement. The maturity assessment laid the foundation for sites to identify and prioritize key areas to advance clinical trial diversity capabilities, and each has made tangible progress. In parallel to completing the assessment with these early sites to understand their maturity and set actionable goals, we also collected their feedback on content validity (e.g., clarity, comprehensiveness, terminology) and feasibility (e.g., ability to collect needed information and data, time required). We describe refinements made to improve the assessment and streamline the process. The EQBMED program will deploy the assessment across various site types (e.g., FQHCs, safety net hospitals) and make further refinements as warranted.

**Conclusions:**

Strategic investment in clinical trial diversity requires structured assessment of site maturity as a starting point for collaborative action. We propose the EQBMED maturity model as a first step toward informing efforts to increase representation of diverse populations in clinical research.

**Supplementary Information:**

The online version contains supplementary material available at 10.1186/s13063-024-08594-9.

## Background

Although efforts to increase diversity in clinical trials have been underway for decades [1], only recently has eliminating inequities in access to clinical trials been defined as a national priority [2–6], with substantial investments from multiple sectors [3, 7–10]. Achieving meaningful gains toward this ambitious goal will require large shifts in workforce, trial design and regulatory arenas [7, 11, 12], as well as alignment of a wide array of competing interests and incentives [13, 14].

Among the most powerful barriers to broader inclusion of communities of color in clinical trials are social determinants of health [15], trustworthiness of health care providers and research institutions [11, 16], and competing pressures on potential participants [17–21]. Despite this evidence, current tools to assess organizational capabilities for clinical trial diversity focus primarily on trial infrastructure, rely solely on quantitative self-reported data, and do not include meaningful assessment of capabilities related to community engagement [22–25]. Recent guidance including a toolkit with logic models aimed to help operationalize guidance with clearly defined scopes for distinct areas of focus; however, as stated in the guide, logic models may overlook intersectionality between interrelated domains of the clinical research enterprise, within a particular organization or between organizations [10]. The Clinical Trials Transformation Initiative (CTTI) [26, 27] model provides an important foundation, yet does not fully operationalize measurement of core concepts and define maturity levels for practical use by sites and partners seeking to improve clinical trial diversity.

One increasingly popular tool for assessing an organization’s current (and aspirational) capabilities and mapping a logical path from initial state to full maturity with regard to a specific goal is the maturity model [28, 29]. Maturity models can be used as a diagnostic tool to determine current capacity across specified domains, prioritize areas for improvement and growth, and serve as a benchmark for an organization to track progress toward their maturity goals [30–32]. In the context of clinical trials, a maturity model can identify needs and assets across highly varied types of trial sites and inform peer mentoring approaches in which sites share complementary strengths in order to progress toward defined maturity goals.

**Fig. 1:**
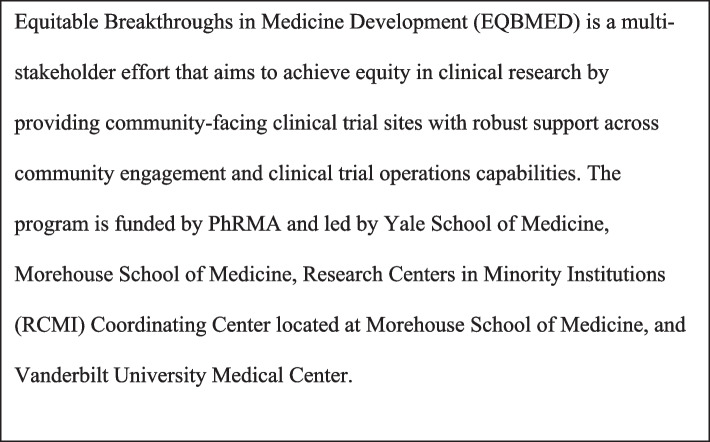
Equitable Breakthroughs in Medicine Development

As part of the Equitable Breakthroughs in Medicine (EQBMED) initiative (Fig. 1), we sought to address the limitations of prior assessment tools and harness the potential of a maturity model approach by developing the EQBMED Site Maturity Model and associated assessment. The assessment is a holistic, collaborative, site-driven formative process carried out with clinical trial sites to catalog their current capabilities and identify opportunities for growth in both conducting industry-sponsored clinical trials and enriching diversity of those trials. The assessment is not meant to be evaluative in nature, or to be used to compare or benchmark sites against others for two reasons. First, the assessment is site-driven, formative and focused on informing context-specific actions at the site. Second, clinical trial sites are becoming increasingly diverse, particularly with innovations in decentralized approaches. While this diversity is potentially quite powerful, it presents challenges in development of universal benchmarking standards, which have not yet been developed. Here we describe the maturity model and associated assessment, the methodology we used in its development, and how findings can support clinical trial diversity efforts. The complete EQBMED Maturity Assessment Site Guide, Site Questionnaire and Rubric documents can be accessed at: https://eqbmed.org/innovations/.

## Methods

**Fig. 2:**
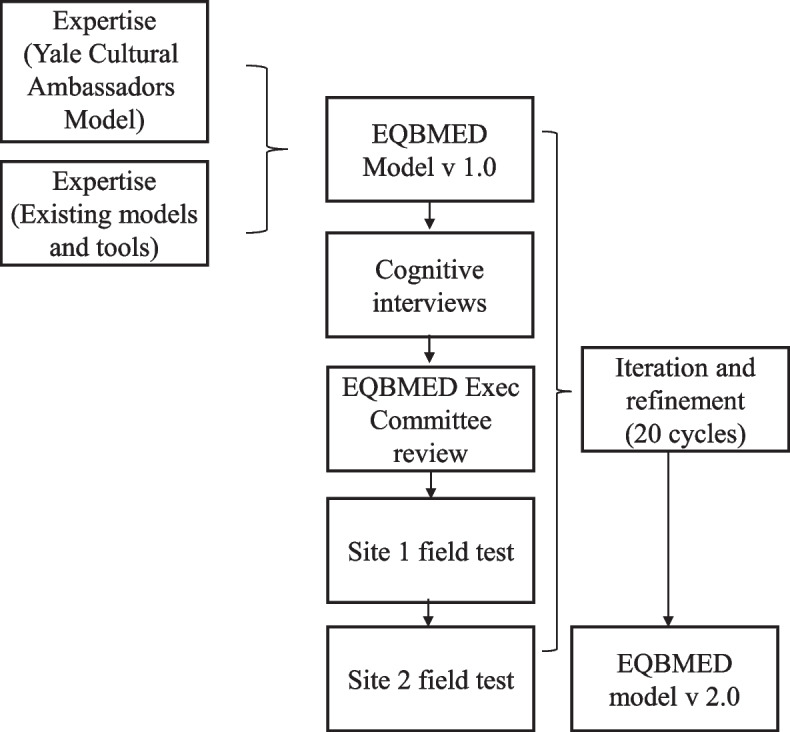
EQBMED Maturity Model Development Process

In developing the maturity model, we drew upon multiple sources of input. The authors, together with the EQBMED team, reviewed, synthesized, and incorporated learnings from the following sources (Fig. 2). In total, there were 20 iterations of the model throughout the development and piloting phases.

1. The Yale Cultural Ambassadors Model's successful, 15-year maturity journey to promote diversity and inclusion in clinical research [33–35]. Success is evidenced by increasing underrepresented communities of color participation in clinical trials from approximately 3% in 2010 to rates now close to 35%, with studies engaging the Cultural Ambassadors directly having rates averaging around 62% and retention rates averaging 97%.x.
2. Comprehensive library of guidelines, principles, toolkits from the CTTI [26, 27], National Academy of Sciences [5, 36], Pharmaceutical Research and Manufacturers of America Pharmaceutical Research and Manufacturers of America (PhRMA) [37], Food and Drug Administration (FDA) [38], Multi-Regional Clinical Trials (MRCT) Centers [39, 40], and others [23, 32, 41].
3. Over a dozen content experts representing decades of expertise and experience in clinical trial operations, pharmaceutical companies, federal regulations, community engagement, organizational readiness, and maturity model building. Feedback revealed the need to ensure robust quantitative and qualitative measurement and supporting documentation; broaden barriers beyond social determinants of health; reduce redundancy across components; clarify distinctions between institutional diversity, equity and inclusion and trial diversity efforts; and bring precision to the community engagement domain.
4. Modified cognitive interviews to test content validity and feasibility at one trial site, and user feedback from full field administration at three trial sites. The cognitive interview guide was designed to elicit critical input from end users of the maturity model. For each subcomponent, we asked: Is the terminology clear? Any suggestions for rephrasing or things to drop? What are some potential sources of insight/documentation? Do you have these sources or can you get them? How difficult or easy is it to access supporting documents? Are there better sources of insight for these subcomponents? The structured tool is included as Supplemental File 1.

### The EQBMED Site Maturity Model

**Fig. 3:**
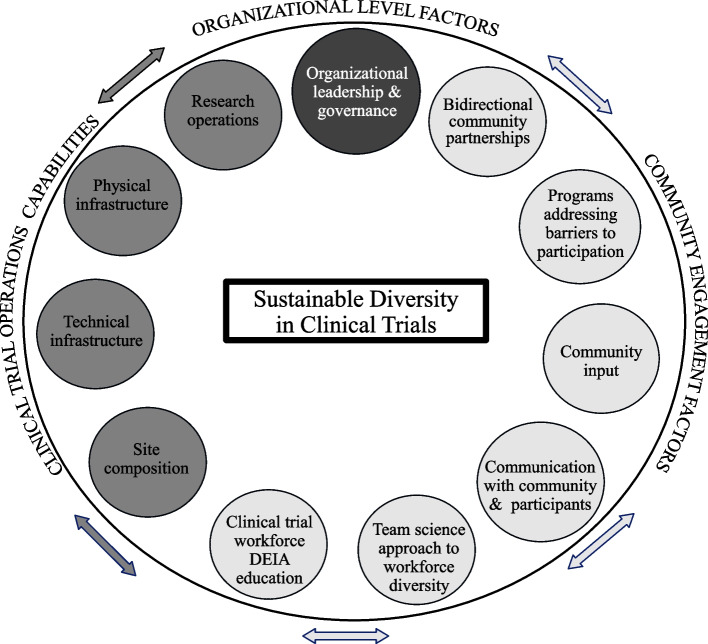
EQBMED Site Maturity Model

The EQBMED Site Maturity Model is shown in Fig. 3. The model consists of 11 components within three domains: (1) organizational level factors, (2) community engagement factors, and (3) clinical trial operations capabilities. When taken together, these components provide a comprehensive description of a site’s maturity in terms of clinical trial diversity. Each component includes 2–7 questions (54 questions in total), accompanied by a rubric to capture maturity for each question and component. Detailed definitions of each component appear in Table 1.

**Table 1:** Component definitions

Organizational level factors	
Organizational leadership and governance	Senior leadership commitment to enhancing diversity in clinical trials, as demonstrated by: diversity and inclusion-focused organizational values; allocation of resources aligned with these organizational values; structures and policies that support goal setting, performance management tracking and accountability for clinical trial diversity; strategic planning to ensure long term sustainability of trial diversity efforts; visible endorsement and participation in programs and activities directed at trial diversity
**Community engagement factors**	
Bi-directional community partnerships	Active collaborations in which all partners have some share of ownership, decision-making, development, and promotion of programs to support clinical trial diversity. [Note: partnerships may address broader organizational goals rather than clinical trials diversity per se]
Programs to address barriers to recruitment and retention	Programs and resources that address economic barriers to participation in clinical trials (e.g., insurance, housing, employment benefits, childcare, transportation, nutritional supports), as well as social and cultural barriers to engagement (e.g., distrust of the healthcare system, lack of cultural humility of staff and investigators, bias leading to not being asked to participate)
Community input to trial design and implementation	Policies and practices that empower community members to provide input to trial design, recruitment, and retention as well as research engagement approaches. Mechanisms may include ethics committees that are prepared to address issues related to recruitment of diverse populations (e.g., coercion, inadequate disclosures), community representation on Institutional Review Boards and community studios
Communications with community and clinical trial participants	Capabilities that ensure accurate, culturally tailored, meaningful written and verbal communications between investigators, community members, and trial participants. Translation, interpretation, and communication services provide all trial-related materials in participants’ preferred language, using standard techniques for addressing literacy, numeracy, cultural framing at every point of contact
Team science approach to clinical trial workforce and leadership diversity	Programs to improve Black, Hispanic, & Latino representation among clinical trial staff, developed and implemented in collaboration with community partners. Formal mentoring, training, and other resource supports are available within the organization for diverse staff, with clearly defined career ladders and opportunities for professional advancement
Clinical trial workforce diversity, equity, inclusion, and accessibility (DEIA) education	Education and training for staff and investigators that enable them to understand and apply principles and practices of DEIA and cultural competency. Programs may include specific content on clinical trials, and community experiences for (e.g., volunteering). Organizations may measure DEIA/cultural competency/humility and use this information for coaching and professional development
**Clinical trial operations capabilities**	
Site composition	A broad array of features of the trial site including overall structure (e.g., secondary sites, partnerships with healthcare organizations), focal therapeutic areas, trial workforce size and experience, finance management and regulatory capabilities, investments in staff training
Technical infrastructure	Information technology to support the conduct of clinical trials (e.g., trial management systems), electronic data capture, electronic health record access for feasibility, recruitment and retention, source data collection, billing segregation, reporting, and post-study follow-up
Physical infrastructure	Physical space and facilities to support the conduct of clinical trials (e.g., storage, for research team), ancillary services (e.g., laboratory, imaging, investigational pharmacy and device management) and equipment (e.g., centrifuge, weight & height scale, refrigerator, freezer, compounding)
Research operations	A broad array of capabilities to support the conduct of clinical trials (e.g., human resource functions, SOPs, IRB, contracting, performance monitoring, quality assurance)

### Completing the assessment

The process for completing the assessment was designed to encourage small group collaboration and open discussions, minimize site burden in completing the assessment, and generate information related to a site’s maturity in specific areas that support clinical trial diversity. There are 3 major steps in completing the site maturity assessment:

#### Step 1: Inventory site capabilities

A core team of site representatives should include individuals able to speak at a high level about all site activities and programming (i.e., community engagement, ongoing trials, and clinical trial governance and organizational leadership), as well as those with expertise in clinical trial operations and community engagement capabilities. After an initial introduction to share an overview of the purpose of the assessment and what to expect, the team convenes in 2–3 meetings. The assessment team supports site representatives in completing the assessment, answering all questions for each component, and noting if a specific question is not applicable to the site and the rationale for why it is not relevant. The assessment team and site representatives work together to determine if any supplemental information or documents are needed or would be helpful in developing the site maturity roadmap (e.g., annual reports, budget information, descriptions of community partnerships, standard operating procedures (SOP) manuals, and clinical trial experience).

#### Step 2: Assign maturity levels

**Fig. 4:**
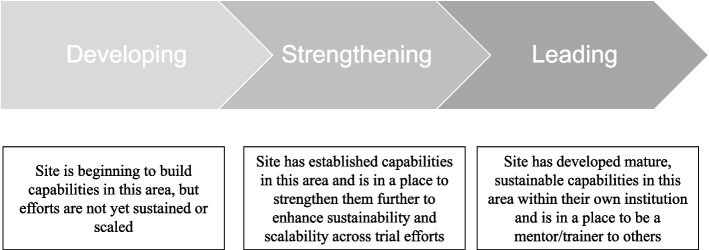
Maturity levels

The model defines three levels of maturity (Fig. 4): (1) developing, (2) strengthening, and (3) leading. Importantly, maturity levels are specific to components and subcomponents, are dynamic and are intended to be tracked over time as sites build and strengthen various capabilities. Question-level results are rolled up into a component-level score according to these criteria. The criteria are intended to set a very high bar for “leading” classification and be highly sensitive to “developing” responses so that capacity needs are not underestimated. We made an intentional decision not to assign weights to individual items, as composite items are not equally important/potentially influential, nor are the components equally important/potentially influential in clinical trial diversity performance.

- ▪ If 100% of the responses are in the “leading” level, the site will be described as “leading” for that component.
- ▪ If 50–99% of the responses are in the “strengthening” or “‘leading” level, the site will be described as “strengthening” for that component.
- ▪ If less than 50% of the responses are in the “strengthening” or “leading” level, the site will be described as “developing” for that component.

#### Step 3: Refine and apply results

Findings from the assessment can be used in several ways depending on the unique goals of each site. The assessment is intended to be used as a diagnostic tool to determine current capacity across specified domains, prioritize areas for improvement and growth, and serve as a benchmark for organizational to track progress toward their maturity goals [30–32]. Importantly, because it is site-driven, context-specific, and descriptive in nature, the assessment is not to be used prescriptively or for comparisons. Assigned maturity levels can be shared with internal and external stakeholders to guide strategic planning and investments [30, 41]. In the context of clinical trials, this assessment will identify needs and assets across highly varied types of trials sites and inform peer mentoring approaches in which sites share complementary strengths in order to progress toward their defined maturity goals. Insights can be used to inform:

- *▪ Goal setting*: These growth opportunities may be translated into action-oriented goals specific to each site. Goals may range in focus and scope (e.g., increase staff to expand trial capacity, develop mechanisms to receive and act on community and patient feedback, develop community advisory boards to co-develop site priorities) and will be unique to each site, even for those with similar maturity levels. Goal setting might also include identifying needed resources and supports.
- *▪ Impact metrics*: Once goals are developed, sites may consider developing impact measures to track progress and change over time (e.g., near-term (6–12 months) and long-term (1 + year) measures).
- *▪ Roadmap*: To achieve these goals and impact measures, sites may consider developing a ~ 6–12-month roadmap of key milestones and activities.
- *▪ Re-assessment*: The assessment and these goals should not be static, but rather should be revisited over time as the site achieves goals and matures.

As a process likely embedded within other institutional activities, the comprehensive and context-driven site maturity assessment process may also reveal insights and actionable strategies that enhance clinical trials work beyond a site’s EQBMED activities. An illustrative example of a completed assessment is provided in Supplement 2.

Several important assumptions underpin the guiding model. First, organizational readiness to engage in authentic, sustainable clinical trial diversity initiatives is a highly complex, multifaceted phenomenon including both technical and relational dimensions [42, 43]. As such, assessment of organizational readiness requires a mixed methods approach using both quantitative and qualitative measures [44]. Robust quantitative measures (e.g., number and types of trials, enrollment, and retention data) are needed to develop key performance indicators to track progress along the model levels. Qualitative data (e.g., notes and transcripts of interviews) characterize other essential capabilities to achieve clinical trial diversity such as the nature of community partnerships and the commitment of senior leadership. Second, the process of assessment must be collaborative in nature for several reasons, including ensuring reliability and validity of the inputs and fostering ownership of the results among the sites. Productive, meaningful collaboration requires trust among partners. Trust is facilitated by investing time and good will in relationship building, creating conditions that encourage candid reflection and exchange by both parties, and deferring to the site representative team to define their aspirational goals. While perhaps not feasible within the current EQBMED Learning Phase, ideally assessments would take place on site, in person. Requiring supporting “evidence” of various site capabilities does not engender trust and should be done with emphasis on developing a shared understanding of maturity status, rather than an “audit” function. Finally, the model reflects unification of two components—research operations and community engagement—since sustainable clinical trial diversity efforts must overcome these silos. Accordingly, the assessment must have input from a range of organizational representatives, including clinical trial staff, investigators, and senior leadership across operations and community engagement.

## Results

A key step in the development, iteration, and testing of the assessment was deploying the tool at sites with varying levels of maturity in clinical trials and community engagement. The first three sites were diverse in size (e.g., < 250–1 K beds), with varying levels of clinical trial capabilities and community engagement. Completing the initial assessment required ~ 4–7 working sessions over the course of ~ 8–10 weeks across the sites, in addition to offline data gathering (e.g., information on staff demographics, trial volume by phase) estimated to have taken ~ 6–10 h. An individual was appointed as “site lead” to complete the assessment and was tasked with engaging relevant experts across clinical trial operations and community engagement topics as needed, with ~ 5–10 additional experts engaged in total for each site. The process began with a kick-off meeting where we introduced the maturity model and objectives, discussed the site’s current state and high-level aspirations, and aligned on next steps for completion, focusing on one factor at a time (e.g., clinical trial operations, community engagement). The assessment culminated in identification of several strengths and areas of opportunity identified across both clinical trial operations (e.g., installing a clinical trials management system (CTMS), augmenting staff capacity) and community engagement (e.g., creating opportunities for earlier engagement with community members to inform research priorities) for each of the sites.

Against the identified areas of opportunity, each site engaged in additional working sessions to identify and prioritize concrete goals, and for each goal, a set of Key Performance Indicators (KPIs) to track progress and impact (e.g., # trials selected based on community-asserted research priorities, # new trials activated, % active trials transitioned to CTMS, completion of an audit-readiness checklist). Since the assessment and identification of these goals and KPIs, the sites have made significant progress in their maturity to conduct diverse clinical trials. For example, one site has taken on 7 new clinical trials, implemented a new quality assurance initiative, installed a CTMS system, identified individuals that will serve as their Community Advisory Board, and more. Another EQBMED site, early in their clinical trials journey, has hired a research coordinator, defined their internal governance across stakeholders to engage as clinical trial leaders, and more. Another EQBMED site looking to expand the volume and reach of their clinical trials has focused on training more staff and installing a CTMS system. Early on, sites have made observations about using the assessment process and tool to simply highlight the complexity of the clinical trials diversity work and how many different parties need to be involved/committed and being able to differentiate or identify small steps versus larger steps that could be implemented to advance their clinical trials work. The assessment has laid the foundation for these sites to identify and prioritize key areas to advance clinical trial diversity capabilities, and each has made tangible progress. The impact of this assessment across these sites and others underway has solidified our confidence in its validity and usefulness. The EQBMED program continues to deploy the assessment across a variety of site types (e.g., FQHCs, safety net hospitals).

### Learnings and revisions to the model

Since this early deployment of the maturity model, we have made several refinements to improve the assessment and streamline the process. In parallel to completing the assessment with these early sites to understand their maturity and set actionable goals, we also collected their feedback on content validity (e.g., clarity, comprehensiveness, terminology) and feasibility (e.g., ability to collect needed information and data, time required). This led to changes such as the addition of “organizational-level factors” as a core category to ensure a more comprehensive view of how clinical trial diversity aligned with leadership and governance at each site. In addition, several granular changes were made to further clarify terminology, simplify criteria, and ensure comprehensiveness. For example, we refined “leading” and “strengthening” classifications for community engagement, differentiating frequent community engagement and interaction from actual participation and leadership in shaping the research processes. Beyond testing and optimizing content, several changes were made to streamline the process and minimize site burden. In our initial deployment of the assessment at sites, uncertainty regarding which site representatives to engage and data needs, competing organizational priorities, and other factors extended the timeline for completion. We have now streamlined the assessment where possible. For example, the process begins with an introductory video explaining the assessment and its purpose, identifying key experts to engage, and outlining how to move forward, followed by just 2–3 working sessions and asynchronous collaboration. With this new process no more than ~ 4–6 h of active time are required to complete the assessment for the site and assessors, although there may be additional time associated with tracking down information as sites prepare their assessment responses. Ultimately, the time required to complete the assessment will depend on the site—their competing organizational priorities, availability of key information, and accessibility of experts—however, our early iterations and learnings with sites have enabled us to streamline the process.

### Limitations

The assessment has several limitations. First, while taxonomy categories should be mutually exclusive and substantively exhaustive as feasible, interrelatedness is unavoidable given the complexity of organizational capacity for achieving clinical trial diversity. Second, it is possible that an important aspect of organizational readiness has not been included. However, the assessment was developed following design thinking principles, including input of end users, other experts, iterative refinement, and field testing [45], along with learnings from Yale Cultural Ambassador’s maturity journey, in order to ensure a comprehensive diverse set of inputs into content domains to the greatest extent possible. Third, there are no universally accepted standards for validating maturity models. [45] However, we used multiple common methods of validation including iterative review by over 20 domain experts, 2 waves of modified cognitive interviews [46] to assess content validity [30] (clarity, comprehensiveness) and feasibility (ability to assemble artifacts, supporting evidence, time required to complete), and piloting in “real environments” to gather user feedback, test applicability, and inform refinements [45]. Iteration is key in the development and validation process. There have been 20 iterations on the assessment to date, including some with more granular categories for maturity level. It is our hope that by sharing this assessment with the broader clinical trial ecosystem beyond EQBMED stakeholders, further iterations can be developed with improvements based on a broader set of real-world learnings. Fourth, patients and members of the public were not involved in the direct development of the assessment; however, elements of the assessment and supporting documents have been informed by more than a decade of work with the Yale Cultural Ambassadors and nearly two decades of a community-academic partnership facilitated by the Yale School of Medicine. EQBMED is also guided by an Advisory Committee comprised of patients, members of the public, and advocacy groups who offered guidance on some of the subcomponent questions, particularly in the community engagement and diversity enrichment component. This guidance triangulated with the team’s plan, as their ideas were similar to what the team had considered.

Finally, this model was built for the Learning Phase of EQBMED, which focused on Black and Hispanic/Latino populations. There are many differing dimensions of diversity (e.g., sexual and gender minority status, rural residency, limited English proficiency, age, disability, socioeconomic, other communities of color), as well as intersections among these dimensions.

Many groups are currently underserved in clinical trials, including those sharing characteristics such as demographic factors (e.g., ages under 18 and over 75), social and economic factors (e.g., asylum seekers, incarcerated individuals), and health status (e.g., cognitively impaired, pregnant women). Future work with the maturity model beyond the EQBMED Learning Phase would prioritize addressing unique issues among other underrepresented groups [47].

### Future work

Ongoing and future work with the EQBMED Site Maturity Model will include (1) monitoring progress across early sites; (2) capturing site use and utility of the assessment model and process in their development; (3) adapting the assessment for smaller community sites; and (4) launching a sponsor front door. At the time of manuscript submission there are 7 sites enrolled in EQBMED. Each site will continue collaborating with EQBMED team as they pursue clinical trial diversity goals developed through the assessment, tracking, and reporting KPIs.

In terms of adaptation, the EQBMED Site Maturity Model was indeed developed with a broad range of research sites in mind, including both large academic institutions and smaller community-based research sites. Now that the assessment has been deployed at a range of sites, current and future work will focus on both scalability and flexibility to adapt for different sizes and types of trial partner organizations. Adapted versions would focus on key components most relevant to these sites and provide a more streamlined approach to assessment, while still capturing essential aspects of site maturity in clinical trial diversity. Additionally, we plan to offer resources and support to help smaller sites integrate the tool into their operations effectively. By adapting the tool to different contexts and providing targeted assistance, we aim to ensure that it can be a valuable resource across the spectrum of research settings.

Finally, development of EQBMED's 'sponsor front door' is underway. The sponsor front door is an innovation from the EQBMED program, creating a seamless mechanism for connecting community-based sites and sponsors, providing sponsor opportunities to receive feedback on their protocols, and empowering community-based sites to hold more trials. This involves developing profiles for sites in the EQBMED program (e.g., overviewing site capabilities and demographics, filming facilities, highlighting therapeutic areas of interest to the community, and more) to allow sponsors to quickly assess high-level initial feasibility for trial consideration and reduce site burden. The EQBMED site assessment serves as the foundation for understanding each site’s capabilities and informs site profiles. Although the assessment is site-driven, we foresee that sponsors may increasingly recognize the value of the EQBMED assessment and potentially incorporate it into their site selection criteria. This could lead sponsors to require or strongly encourage sites to complete an EQBMED assessment to demonstrate their readiness and commitment to diverse recruitment efforts. Ultimately, the EQBMED Site Maturity Model aims to facilitate a more equitable approach to clinical trial participation, benefiting both sites and sponsors in achieving more representative trial populations.

## Conclusion

Leveraging increasing strategic investment in clinical trials diversity requires structured assessment of site maturity as a starting point for collaborative action. The development of a readiness assessment to gauge organizational maturity is a first step toward increasing representation of diverse populations in clinical research. We built this assessment as a collaborative effort among community-based sites; EQBMED leaders with decades of experience spanning industry, academia, and the full clinical research ecosystem; experts in clinical trial diversity assessments; scientists specialized in the development of maturity model tools; and scientists specialized in clinical trials, partnership development, social determinants of health, and community engagement. The assessment was conceptualized and implemented as a site-partnered process to elicit a comprehensive set of considerations known to be important to sites caring for underrepresented populations of color. Assets, challenges, and opportunities identified by the assessment can guide implementation solutions most relevant and appropriate for the proposed trial context.

## Supplementary Information

Supplementary Material 1.

Supplementary Material 2.

## Data Availability

Not applicable.

## Abbreviations

CTMS: Clinical Trials Management System
CTTI: The Clinical Trials Transformation Initiative
DEIA: Diversity, Equity, Inclusion, and Accessibility
EQBMED: The Equitable Breakthroughs in Medicine
FDA: Food and Drug Administration
KPIs: Key Performance Indicators
MRCT: Multi-Regional Clinical Trials
PhRMA: Pharmaceutical Research and Manufacturers of America
SOPs: Standard Operating Procedures
YCCI: Yale Center for Clinical Investigation

## References

[1] Swanson GM, Ward AJ. Recruiting minorities into clinical trials toward a participant-friendly system. J Natl Cancer Inst. 1995;87(23):1747–59. 10.1093/jnci/87.23.1747. 7473831 10.1093/jnci/87.23.1747

[2] Organizing Committee for Assessing Meaningful Community Engagement in Health and Health Care Programs and Policies. Assessing meaningful community engagement: a conceptual model to advance health equity through transformed systems for health. NAM Perspect. 2022;22(2):1–11. 10.31478/202202c. 10.31478/202202c PMC9303007 35891775

[3] PhRMA. PhRMA principles on conduct of clinical trials. 2020 April 1, 2024. Available from: https://phrma.org/resource-center/Topics/Cost-and-Value/PhRMA-Principles-on-Conduct-of-Clinical-Trials. Cited 2022 October 28.

[4] U.S. Department of Health and Human Services, Food and Drug Administration, Center for Drug Evaluation and Research (CDER), Center for Biologics Evaluation and Research (CBER). Enhancing the diversity of clinical trial populations – eligibility criteria, enrollment practices, and trial designs guidance for industry 2020 April 1, 2024. Available from: https://www.fda.gov/media/127712/download. Cited 2022 October 22.

[5] National Academies of Sciences E, Medicine, Policy, Global A, Committee on Women in Science E, Medicine, et al. Improving representation in clinical trials and research: building research equity for women and underrepresented groups. Alex H, Kirsten B-D, editors. Washington, D. C.: National Academies Press; 2022. 36137057

[6] U.S. Government Accountability Office. Cancer clinical trials: federal actions and selected non-federal practices to facilitate diversity of patients. GAO-23–1052452022 April 1, 2024. Available from: https://www.gao.gov/products/gao-23-105245.

[7] Olson S, Anderson KM, Board on Population H, Public Health P, Health, Medicine D, et al. Strategies for ensuring diversity, inclusion, and meaningful participation in clinical trials: proceedings of a workshop. 1 ed. Steve O, Karen MA, editors. Washington, D. C.: National Academies Press; 2016. 27606381

[8] Nexus Community Partners. Community engagement assessment tool 2018 Ap 1, 2024. Available from: https://www.nexuscp.org/wp-content/uploads/2017/05/05-CE-Assessment-Tool.pdf. Cited 2022 October 28.

[9] 9. Deloitte Center for Health Solutions, Pharmaceutical Research and Manufacturers of America (PhRMA). Enhancing clinical trial diversity: stakeholder perspectives on advancing research through representative clinical trials. 2021 April 1, 2024. Available from: https://www2.deloitte.com/us/en/insights/industry/life-sciences/lack-of-diversity-clinical-trials.html. Cited 2022 October 28.

[10] The MRCT Center of Brigham and Women's Hospital and Harvard. Achieving diversity, inclusion and equity in clinical research: toolkit 2021 April 1, 2024. Available from: https://mrctcenter.org/diversity-in-clinical-research/tools/toolkit/. Cited 2022 October 28.

[11] Gaddy JJ, Gross CP. Addressing racial, ethnic, and age disparities in cancer clinical trial enrollment: time to stop tinkering around the edges. JAMA Oncol. 2022;8(12):1792–93. 10.1001/jamaoncol.2022.5006. 10.1001/jamaoncol.2022.5006 36301581

[12] Kelsey MD, Patrick-Lake B, Abdulai R, Broedl UC, Brown A, Cohn E, et al. Inclusion and diversity in clinical trials: Actionable steps to drive lasting change. Contemp Clin Trials. 2022;116:106740. 10.1016/j.cct.2022.106740. 10.1016/j.cct.2022.106740 PMC9133187 35364292

[13] Hwang TJ, Brawley OW. New Federal Incentives for Diversity in Clinical Trials. N Engl J Med. 2022;387(15):1347–9. 10.1056/NEJMp2209043. 36214598 10.1056/NEJMp2209043

[14] Varma T, Jones CP, Oladele C, Miller J. Diversity in clinical research: public health and social justice imperatives. J Med Ethics. 2023;49(3):200–3. 10.1136/medethics-2021-108068. 35428737 10.1136/medethics-2021-108068

[15] Unger JM, Vaidya R, Hershman DL, Minasian LM, Fleury ME. Systematic review and meta-analysis of the magnitude of structural, clinical, and physician and patient barriers to cancer clinical trial participation. J Natl Cancer Inst. 2019;111(3):245–55. 10.1093/jnci/djy221. 30856272 10.1093/jnci/djy221 PMC6410951

[16] Jaiswal J. Whose responsibility is it to dismantle medical mistrust? Future directions for researchers and health care providers. Behav Med (Washington, DC). 2019;45(2):188–96. 10.1080/08964289.2019.1630357. 10.1080/08964289.2019.1630357 PMC7808309 31343959

[17] Borno HT, Andemeskel G, Palmer NR. Redefining attribution from patient to health system—How the notion of “Mistrust” places blame on black patients. JAMA Onco. 2021;7(5):780. 10.1001/jamaoncol.2020.8482. 10.1001/jamaoncol.2020.8482 33662096

[18] Brincks AM, Shiu-Yee K, Metsch LR, del Rio C, Schwartz RP, Jacobs P, et al. Physician mistrust, medical system mistrust, and perceived discrimination: associations with HIV Care Engagement and Viral Load. AIDS Behav. 2019;23(10):2859–69. 10.1007/s10461-019-02464-1. 30879211 10.1007/s10461-019-02464-1 PMC6854532

[19] Fisher JA. Institutional mistrust in the organization of pharmaceutical clinical trials. Med Health Care Philos. 2008;11(4):403–13. 10.1007/s11019-008-9154-y. 18633728 10.1007/s11019-008-9154-y PMC2952304

[20] Gray Ii DM, Nolan TS, Bignall ONR, Gregory J, Joseph JJ. Reckoning with our trustworthiness, leveraging community engagement. Popul Health Manag. 2022;25(1):6–7. 10.1089/pop.2021.0158. 34271849 10.1089/pop.2021.0158

[21] Pahus L, Suehs CM, Halimi L, Bourdin A, Chanez P, Jaffuel D, et al. Patient distrust in pharmaceutical companies: an explanation for women under-representation in respiratory clinical trials? BMC Med Ethics. 2020;21(1):72. 10.1186/s12910-020-00509-y. 32791969 10.1186/s12910-020-00509-y PMC7424561

[22] Dimond EP, Zon RT, Weiner BJ, St Germain D, Denicoff AM, Dempsey K, et al. Clinical trial assessment of infrastructure matrix tool to improve the quality of research conduct in the community. J Oncol Pract. 2016;12(1):63–4 e23–35. 10.1200/JOP.2015.005181. 10.1200/JOP.2015.005181 PMC4976452 26627979

[23] Foster D. The Diversity Site Assessment Tool (DSAT), reliability and validity of the industry gold standard for establishing investigator site ranking. Integr J Med Sci. 2020;7:1–13. 10.15342/ijms.7.266.

[24] Andrasik MP, Broder GB, Wallace SE, Chaturvedi R, Michael NL, Bock S, et al. Increasing black, indigenous and people of color participation in clinical trials through community engagement and recruitment goal establishment. PloS One. 2021;16(10):e0258858. 10.1371/journal.pone.0258858 PMC8525736 34665829

[25] de las Nueces D, Hacker K, DiGirolamo A, Hicks LS. A systematic review of community-based participatory research to enhance clinical trials in racial and ethnic minority groups. Health Serv Res. 2012;47(3 Pt 2):1363–86. 10.1111/j.1475-6773.2012.01386.x. 10.1111/j.1475-6773.2012.01386.x PMC3418827 22353031

[26] Corneli A, Hallinan Z, Hamre G, Perry B, Goldsack JC, Calvert SB, et al. The clinical trials transformation initiative: methodology supporting the mission. Clin Trials (London, England). 2018;15(1_suppl):13–8. 10.1177/1740774518755054. 10.1177/1740774518755054 29452520

[27] Corneli A, Hanlen-Rosado E, McKenna K, Araojo R, Corbett D, Vasisht K, et al. Enhancing diversity and inclusion in clinical trials. Clin Pharmacol Ther. 2023;113(3):489–99. 10.1002/cpt.2819. 36628990 10.1002/cpt.2819

[28] Iversen J, Nielsen P, Norbjerg J. Situated assessment of problems in software development. ACM SIGMIS Database: DATABASE Adv Inform Syst. 1999;30(2):66–81. 10.1145/383371.383376.

[29] Pöppelbuß J, Röglinger M. What makes a useful maturity model? A framework of general design principles for maturity models and its demonstration in business process management. In the Proceedings of the Nineteenth Eurpoean Converence on Information Systems. Helsinki; 2011. Available at: https://fim-rc.de/Paperbibliothek/Veroeffentlicht/327/wi-327.pdf.

[30] Becker J, Knackstedt R, Pöppelbuß J. Developing maturity models for IT management: a procedure model and its application. Bus Inf Syst Eng. 2009;1(3):213–22. 10.1007/s12599-009-0044-5.

[31] Hevner AR, March ST, Park J, Ram S. Design science in information systems research. MIS Q. 2004;28(1):75–105. 10.2307/25148625.

[32] Solli-Sæther H, Gottschalk P. The modeling process for stage models. J Organ Comput Electron Commer. 2010;20(3):279–93. 10.1080/10919392.2010.494535.

[33] U.S. Food & Drug Administration. MOU 225–18–0152018. Available from: https://www.fda.gov/about-fda/academic-mous/mou-225-18-015.

[34] Public Responsibility in Medicine and Research, editor The Yale Model: Community and Participant Engagement at Advancing Ethical Research Conference Diversity and Inclusion in Clinical Research Session. PRIM&R 2020 Advancing Ethical Research Conference (AER20) Virtual Event; 2020.

[35] Araojo R, Johnson TH, Spinner JR. Advancing clinical trial diversity through community engagement. DIA Global. 2019. Available from: https://globalforum.diaglobal.org/issue/october-2019/advancing-clinical-trial-diversity-through-community-engagement/.

[36] Bibbins-Domingo K, Helman A, Dzau VJ. The imperative for diversity and inclusion in clinical trials and health research participation. J Am Med Assoc. 2022;327(23):2283–4. 10.1001/jama.2022.9083. 10.1001/jama.2022.9083 35579885

[37] Younossi A, Sanhai W, Shah S, Chang C, Overman J. Enhancing clinical trial diversity. 2023. Available from https://www2.deloitte.com/us/en/insights/industry/life-sciences/lack-of-diversity-clinical-trials.html.

[38] U.S. Food and Drug Administration. Clinical trial diversity 2022. Available from: https://www.fda.gov/consumers/minority-health-and-health-equity/clinical-trialdiversity.

[39] Bierer BE, White SA, Meloney LG, Ahmed HR, Strauss DH, Clark LT. Achieving diversity, inclusion, and equity in clinical research guidance document version 1.2.2021 April 1, 2024. Available from: https://mrctcenter.org/diversity-in-clinical-research/wp-content/uploads/sites/11/2021/09/MRCT-Center-Diversity-Guidance-Document-Version-1.2.pdf. Cited 2022 October 28.

[40] Bierer B. E., White S .A., Meloney L. G., Ahmed H. R., Strauss D. H., Clark L. T. Achieving diversity, inclusion, and equity in clinical research toolkit version 1.2. Guidance toolkit & user guide. Cambridge and Boston, MA: Multi-Regional Clinical Trials Center of Brigham and Women’s Hospital and Harvard (MRCT Center). 2021 April 1, 2024. Available from: https://mrctcenter.org/diversity-in-clinical-research/tools/toolkit/.

[41] Röglinger M, Pöppelbuß J. What makes a useful maturity model? A framework for general design principles for maturity models and its demonstration in business process management. In: The proceedings of the nineteenth european conference on information systems (ecis 2011), association for information systems (ais), paper 28. 2011. Available at: https://fim-rc.de/Paperbibliothek/Veroeffentlicht/327/wi-327.pdf.

[42] Stevens GW. Toward a process-based approach of conceptualizing change readiness. J Appl Behav Sci. 2013;49(3):333–60. 10.1177/0021886313475479.

[43] Weiner BJ, Amick H, Lee SYD. Review: conceptualization and measurement of organizational readiness for change: a review of the literature in health services research and other fields. Los Angeles, CA: SAGE Publications; 2008. p. 379–436. 10.1177/1077558708317802 18511812

[44] Curry LA, Nembhard IM, Bradley EH. Qualitative and mixed methods provide unique contributions to outcomes research. Circulation (New York, NY). 2009;119(10):1442–52. 10.1161/CIRCULATIONAHA.107.742775. 10.1161/CIRCULATIONAHA.107.742775 19289649

[45] Wendler R. The maturity of maturity model research: a systematic mapping study. Inf Softw Technol. 2012;54(12):1317–39. 10.1016/j.infsof.2012.07.007.

[46] Willis GB. Cognitive interviewing and questionnaire design: a training manual. Cognitive methods staff working paper series, no. 71994 April 1, 2024. Available from: http://www.srl.uic.edu/links/CMS_WP07_Willis_1994_CogIntTraining.pdf.

[47] National Institute for Health and Care Research. Improving inclusion of under-served groups in clinical research: Guidance from the INCLUDE project. 2020. Available from: https://www.nihr.ac.uk/improving-inclusion-under-served-groups-clinical-research-guidance-include-project. Cited 2020 Month Day.

