# Supplementary material for: Supporting diversity in clinical trials: the equitable breakthroughs in medicine site maturity model
Source: Trials. 2024 Nov 14;25:764. doi: 10.1186/s13063-024-08594-9 (PMC11566401; doi:10.1186/s13063-024-08594-9)
Supplement: Supplementary file 1 — Supplementary Material 1. [file 13063_2024_8594_MOESM1_ESM.pdf]

Figure 5: Process to complete the site maturity assessment

Illustrative example: Process to complete the site maturity assessment

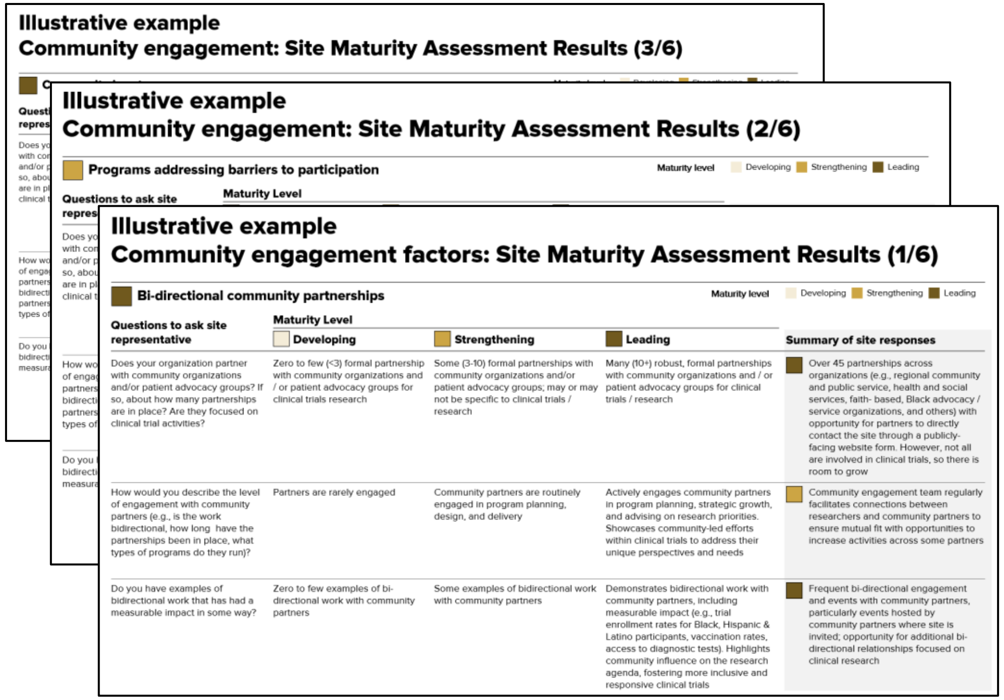

Detailed deep dives of maturity assessment by question

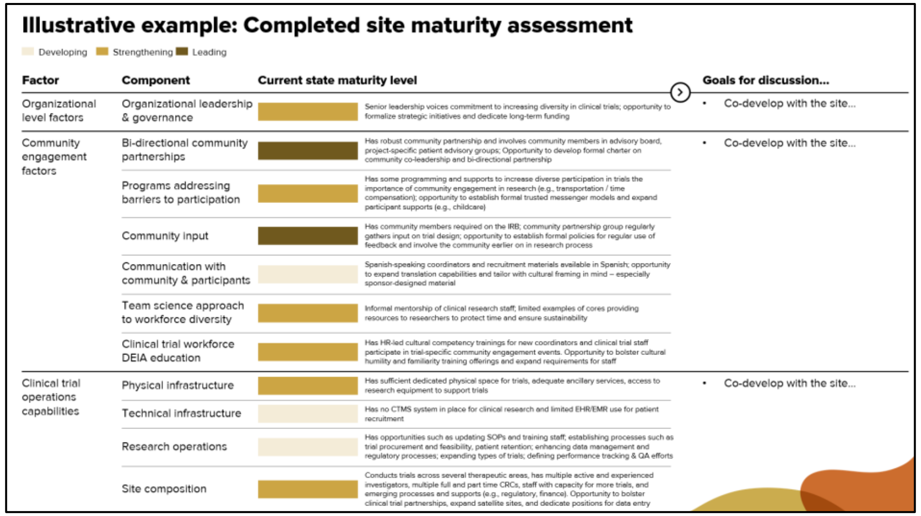

Example synthesis of completed maturity assessment by component, for discussion with site to co-develop goals

Illustrative example: Completed site maturity assessment

Developing | Strengthening | Leading

Factor	Component	Current state maturity level		Goals for discussion…
Organizational level factors	Organizational leadership & governance	Strengthening	Senior leadership voices commitment to increasing diversity in clinical trials; opportunity to formalize strategic initiatives and dedicate long-term funding	• Co-develop with the site…
Community engagement factors	Bi-directional community partnerships	Leading	Has robust community partnership and involves community members in advisory board, project-specific patient advisory groups; Opportunity to develop formal charter on community co-leadership and bi-directional partnership	• Co-develop with the site…
	Programs addressing barriers to participation	Strengthening	Has some programming and supports to increase diverse participation in trials the importance of community engagement in research (e.g., transportation / time compensation); opportunity to establish formal trusted messenger models and expand participant supports (e.g., childcare)	
	Community input	Leading	Has community members required on the IRB; community partnership group regularly gathers input on trial design; opportunity to establish formal policies for regular use of feedback and involve the community earlier on in research process	
	Communication with community & participants	Developing	Spanish-speaking coordinators and recruitment materials available in Spanish; opportunity to expand translation capabilities and tailor with cultural framing in mind – especially sponsor-designed material	
	Team science approach to workforce diversity	Strengthening	Informal mentorship of clinical research staff; limited examples of cores providing resources to researchers to protect time and ensure sustainability	
	Clinical trial workforce DEIA education	Strengthening	Has HR-led cultural competency trainings for new coordinators and clinical trial staff participate in trial-specific community engagement events. Opportunity to bolster cultural humility and familiarity training offerings and expand requirements for staff	
Clinical trial operations capabilities	Physical infrastructure	Strengthening	Has sufficient dedicated physical space for trials, adequate ancillary services, access to research equipment to support trials	• Co-develop with the site…
	Technical infrastructure	Developing	Has no CTMS system in place for clinical research and limited EHR/EMR use for patient recruitment	
	Research operations	Developing	Has opportunities such as updating SOPs and training staff; establishing processes such as trial procurement and feasibility, patient retention; enhancing data management and regulatory processes; expanding types of trials; defining performance tracking & QA efforts	
	Site composition	Strengthening	Conducts trials across several therapeutic areas, has multiple active and experienced investigators, multiple full and part time CRCs, staff with capacity for more trials, and emerging processes and supports (e.g., regulatory, finance). Opportunity to bolster clinical trial partnerships, expand satellite sites, and dedicate positions for data entry	

Illustrative example: Completed site maturity assessment

Developing | Strengthening | Leading

Factor	Component	Maturity level	Goals co-developed with the site
Organizational level factors	Organizational leadership & governance	Strengthening	• Expand leadership capacity to develop an organization-wide strategic plan to advance diversity in clinical trials
Community engagement factors	Bi-directional community partnerships	Leading	• Create opportunities community partners to shape research priorities from the outset<br>• Create formal mentorship and training opportunities for Black, Hispanic & Latino clinical research staff<br>• Consider opportunities to mentor other organizations on community engagement and partnership
	Programs addressing barriers to participation	Strengthening	
	Community input	Leading	
	Communication with community & participants	Developing	
	Team science approach to workforce diversity	Strengthening	
	Clinical trial workforce DEIA education	Strengthening	
Clinical trial operations capabilities	Physical infrastructure	Strengthening	• Integrate clinical research technology into provider workflows to streamline management of clinical research<br>• Formalize research SOPs and establish a streamlined process to manage and update these as needed
	Technical infrastructure	Developing	
	Research operations	Developing	
	Site composition	Strengthening	
