# Supplementary material for: Supporting diversity in clinical trials: the equitable breakthroughs in medicine site maturity model
Source: Trials. 2024 Nov 14;25:764. doi: 10.1186/s13063-024-08594-9 (PMC11566401; doi:10.1186/s13063-024-08594-9)
Supplement: Supplementary file 2 — Supplementary Material 2. [file 13063_2024_8594_MOESM2_ESM.docx]

**EQBMED MATURITY MODEL**

**PILOT TESTING GUIDE**

**11.2.22**

**Instructions**

1. This guide uses a modified cognitive interview approach. The questions and probes are intended to foster collaborative discussion and critical input from end users of the maturity model. Is it clear what the criteria are referring to? Does the language make sense? Are there suggestions for better terminology or definitions?
2. Working together with key representatives from the prospective trial site, review each domain and its subcomponents, assessing both clarity of the content and feasibility of the supporting documentation. Make note of any suggestions or observations, asking clarifying questions as needed.
3. If the site team is working through the model on their own (asynchronously, without the Yale team), they can compile notes and feedback using this document to facilitate their review.

| **Criteria** | **Subcomponents** | **CONTENT: Is the terminology clear? Suggestions for rephrasing or things to add/drop?** | **Potential sources of insight/supporting documentation** | **FEASIBILITY: Do you have these sources, or can you get them? How difficult or easy is it to access supporting documents? Are there better sources of insight for these subcomponents?** |
| --- | --- | --- | --- | --- |
| **Site and scope demographics** | Black, Hispanic, Latino representation across catchment area |  | Census data |  |
|  | Black, Hispanic, Latino representation across patients |  | Administrative data |  |
|  | Clinical trial patient reach |  | Trial data; national population Census data |  |
|  | Specialty reach |  | Trial data; Administrative data |  |
| **Geographic diversity** | New geographic reach from site location |  | Site geographic location/address; Setting classification (e.g., clinical, community, other) |  |
| **Strategic fit** | Strategic fit |  | Signed letter of commitment from CEO/Board Chair; Explicit support for Black, Hispanic & Latino representation in strategic plans; Tracking of trend data on trial data & % Black, Hispanic, Latino representation in clinical trials; Administrative data; Human resource training data; Primary data collection (e.g., interviews/questionnaire |  |
| **Site network** | Partnerships with hubs and local health organizations |  | Administrative data; Publicly available data such as websites; Formal & signed agreements across organizations; Primary data collection (e.g., interviews/questionnaire); Public available online information |  |
|  | Proximity to potential sites |  | Administrative data; Formal & signed agreements across organizations; Primary data collection (e.g., interviews/questionnaire); Public available online information |  |
| **Research capabilities** | Policies and procedures |  | Sponsor, CRO & site SOPs; Procedure documents for SOP updates, compliance & community input |  |
|  | Infrastructure |  | IRB policies & procedures; Budget & resource allocation documents; Site description documents; Program descriptions (for participant supports) w/budget information |  |
|  | Trial and volume experience |  | Trial data; PI CVs; Administrative data on staff training |  |
| **Community**  **engagement** | Community context |  | Systematic stakeholder assessment using established methodologies & tools; Public information related to community trust (e.g. news, other media); Primary data collection (e.g., interview, observation, questionnaire); Assessment completed within the prior 6 months; Program volunteer rosters |  |
|  | Community partnerships |  | MOUs & other signed agreements formalizing partnerships; Partnerships named and described in public documents such as websites; Flyers, reports or other documents describing partnerships |  |
|  | Community co-leadership |  | Signed SOPs and agreements; Clinical trial outreach and recruitment documents; Primary data collection (e.g., interview, observation, questionnaire) |  |
|  | Mechanisms to bring community & study participant voices to study design and implementation |  | Primary data collection (interviews and/or focus groups with site and Black, Hispanic, Latino community members); Documented meetings with Black, Hispanic, Latino community members (e.g., attendance, agendas, action items); Clinical trial policies & procedures; IRB applications; Examples of changes to clinical trial protocols |  |
|  | Capacity for communications with community & study participants including language |  | Staff speaking languages (# & types) determined through HR admin records, interview or questionnaire; Programs & other materials in languages other than English (# & types); Examples of dissemination materials beyond scientific journals |  |
|  | Social determinant of health, other barriers & interventions |  | Reports, journal articles, publicly available information; Primary data collection (interviews, questionnaires, or reports) |  |
| **Technical capabilities** | Performance management capabilities |  | Policies & procedures regarding KPIs; Examples of KPI reports; Policies & procedures regarding QA/QI/audit; Examples of QA/QI/audit reports; Investment in staff capacity to utilize EHR systems (e.g., site training sessions, required demonstration of knowledge through employment requirements) |  |
|  | Technology solutions & clinical research administration systems |  | Policies & procedures regarding EHR & Clinical trial research administration; Investment in staff capacity to utilize EHR systems (e.g., site training sessions, required demonstration of knowledge through employment requirements) |  |
|  | Data capability |  | EDC systems and reports |  |
| **Organizational governance & staff capacity** | Governance |  | Organizational charts; Policies & procedures; Meeting minutes & budget documents |  |
|  | Leadership |  | Written policies; Meeting minutes; Evidence of sharing resources or tools to advance Clinical trial diversity at other organizations seeking to be new sites (e.g., FQHCs); Portfolio of internal communications (newsletters, flyers, emails, meeting minutes) |  |
|  | Staff capacity & education/training (excluding language capacity) |  | Attendance log for DEI training; Required curriculum and hours for DEI training; Primary data collection (interviews & questionnaires); HR records (job descriptions); Evidence of community engagement structures, policies (e.g., office of community engagement) |  |
|  | Workforce diversity |  | Administrative data; Census data; Workforce data (national data on racial & ethnic diversity of principal investigators) |  |
